# Preferential flow pathways in a deforming granular material: self-organization into functional groups for optimized global transport

**DOI:** 10.1038/s41598-019-54699-6

**Published:** 2019-12-03

**Authors:** Joost H. van der Linden, Antoinette Tordesillas, Guillermo A. Narsilio

**Affiliations:** 1 Department of Infrastructure Engineering, The University of Melbourne, Melbourne, Australia; 2 School of Mathematics and Statistics & School of Earth Sciences, The University of Melbourne, Melbourne, Australia

**Keywords:** Applied mathematics, Hydrology, Civil engineering

## Abstract

Existing definitions of where and why preferential flow in porous media occurs, or will occur, assume a priori knowledge of the fluid flow and do not fully account for the connectivity of available flow paths in the system. Here we propose a method for identifying preferential pathways through a flow network, given its topology and finite link capacities. Using data from a deforming granular medium, we show that the preferential pathways form a set of percolating pathways that is optimized for global transport of interstitial pore fluid in alignment with the applied pressure gradient. Two functional subgroups emerge. The primary subgroup comprises the main arterial paths that transmit the greatest flow through shortest possible routes. The secondary subgroup comprises inter- and intra-connecting bridges that connect the primary paths, provide alternative flow routes, and distribute flow through the system to maximize throughput. We examine the multiscale relationship between functionality and subgroup structure as the sample dilates in the lead up to the failure regime where the global volume then remains constant. Preferential flow pathways chain together large, well-connected pores, reminiscent of force chain structures that transmit the majority of the load in the solid grain phase.

## Introduction

Where preferential fluid flow paths in porous media occur, what fraction of flow these paths carry, and why certain pathways are preferred has been defined in various ways, including (1) the delineation of the liquid phase in relatively mobile (preferential) and immobile parts^1^, (2) ‘all phenomena where water and solutes move along certain pathways, while bypassing a fraction of the porous matrix’^2^, [p ∼ 150], and (3) non-equilibrium transport through large interaggregate voids referred to as macropores in soil science^3^. A dedicated body of research has also presented different ways to characterize preferential flow, including dye staining experiments^4,5^, mathematical indicators based on the breakthrough curves and early arrival times^6^, thresholding the highest flow rates (‘hotspots’) in numerical simulations (e.g.^7,8^) and, more recently, complex network approaches focusing on individual paths of least resistance^9–12^. While these studies have continuously improved our understanding of preferential flow, predicting emergent preferential flow remains particularly challenging due to the multi-scale, coupled interplay between complex, heterogeneous pore geometry^13^, capillary, gravity and viscous forces acting on the fluid^14^, pore pressure^15^, antecedent moisture^16^, organic matter^14^ and wettablity effects^17^. Consequently, the emergence of preferential flow is one of the main contributors to uncertainty in the prediction of transport^1,18^. Further progress is impeded by the lack of a quantitative, multi-scale and predictive definition of the structure and location of preferential flow pathways. Indeed, the aforementioned definition (1) and characterization techniques (dye tracing, breakthrough curves and hotspots) cannot predict preferential flow structure without conducting a fluid flow experiment or numerical simulation, while definition (3) does not directly account for the connectivity that is inherent to the chains of pores that make up preferential flow pathways^19^.

Where and why preferential flow paths occur is inherently related to the connectivity and internal properties. Such knowledge plays an important role in a wide range of applications, such as soil contamination^3,13^, fluid homeostasis in the central nervous system^20^, ore dewatering processes^8^, landfill waste leaching^21^ and volcanic lava flow^22^. The size and properties of the fast-flowing, preferential fraction of the pore network within soils, in particular, have repeatedly been highlighted as an important topic of research that has not been conclusively quantified^13,14,23^. Experimental observations point to a complex network of inter- and intra-connected preferential flow segments with varying size and connectivity, rather than disjoint pathways spanning the entire medium, both at the hillslope scale^16^ and in soil columns analyzed using X-ray computerized tomography^19^. Allaire *et al*.^4^ conclude that lateral preferential flow is at least as important as vertical preferential flow (in the direction of gravity) and their interaction is critical for the overall throughput, particularly at large scales, yet these connections are rarely studied. Interconnections between preferential flow paths are also known to be a critical factor for the hydraulic properties of aquifers^24^, and may increase permeability in fractured media with low matrix permeability and high fracture permeability^25^. Connectivity is generally considered one of the most important influencing factors of permeability in porous media^26–31^. Overall, these observations suggest two natural groups within preferential flow paths, namely primary, highway-like preferential route segments, inter- and intra-connected by secondary, bridge-like pathways that support the overall preferential throughput.

Elucidating the functional preferential flow groups requires a mathematical framework that can account for geometrical and material heterogeneity germane to complex porous media. In this context, *network flow theory* and, in particular, the Maximum Flow, Minimum Cost (MFMC) algorithm, has shown promise in modeling (preferential) force transmission^32–34^ in cohesionless and cohesive granular materials in 3D^35,36^. Tordesillas *et al*.^35^ observe a 76–82% capture rate of grains predicted as being ‘preferential’ force transmission pathways by MFMC, measured as a percentage of grains in the (known preferential) force chains (see^37,38^). A similar approach is followed to predict the macro-crack pattern in heterogeneous and multiphase concrete specimens, prior to initiation of actual damage^39^. The use of a network flow model for fluid transmission also has precedent, with Ushizima *et al*.^40^ using the maximum flow component of MFMC to characterize fluid storage after precipitation for carbon sequestration applications. Ju *et al*.^12^ compare shortest paths, used in the minimum cost component of MFMC, to (experimentally) measured preferential flow in two dimensions and observe good agreement in the primary flow pathway structure. Shortest paths are also employed by Rizzo and Barros^11^ to quantify solute transport through heterogeneous porous media, establishing a relationship with the (preferential flow-induced) early arrival times of a solute plume. Further characterization of the optimal flow pathways predicted using a network flow model can be obtained using *complex network theory*. Gao and co-authors, for example, uncover the structure and flow patterns in multi-phase oil-water slug flow using a range of complex network techniques, including PageRank versatility and community structure^41–43^. Given their ability to accurately quantify multi-scale connectivity, analyzing complex systems using complex networks is an increasingly prominent paradigm in numerous scientific areas^44–46^, including the geosciences^31,47^.

In this work, we propose a definition for preferential flow pathways through porous media, as given by the output of the Maximum Flow, Minimum Cost algorithm. We build on the insights gained from applying network flow models to force transmission by applying the algorithm to the network of pores and throats, capturing the domains in which the bulk of fluid flow occurs. MFMC returns a percolating set of optimal pathways through the medium, pushing ‘flow’ in the direction of the pressure gradient. We incorporate both local knowledge of transmission (e.g. Hagen-Poiseuille conductance) and the multi-scale connectivity that is inherent to flow pathways, moving beyond the single-scale views associated with existing definitions. Further building on and formalizing the intuitions behind micropore-macropore and mobile-immobile delineations of the void phase, we employ a statistical model to subdivide preferential pathways into the primary routes and the secondary pathways that connect them. MFMC is applied to a fully saturated deforming granular material, to capture part of the dynamics that are inherent to the manifestation of flow in real (fractured) sands, soils and rocks^48,49^. For a validation of the proposed network flow model on a collection of well-known, static sphere assemblies, we refer to the Supplementary Material. Our physics-based and data-driven model is not intended to replace experimental approaches, such as preferential solute transport imaging using high-resolution X-ray tomography^19,50^, or micromechanical models coupling fluid flow and mechanical deformation^15^. Rather, our network flow model aims to complement such investigations to gain supplemental insights and further our understanding of where, why and to what degree preferential flow paths form in granular, porous media.

## Results

**Figure 1:**
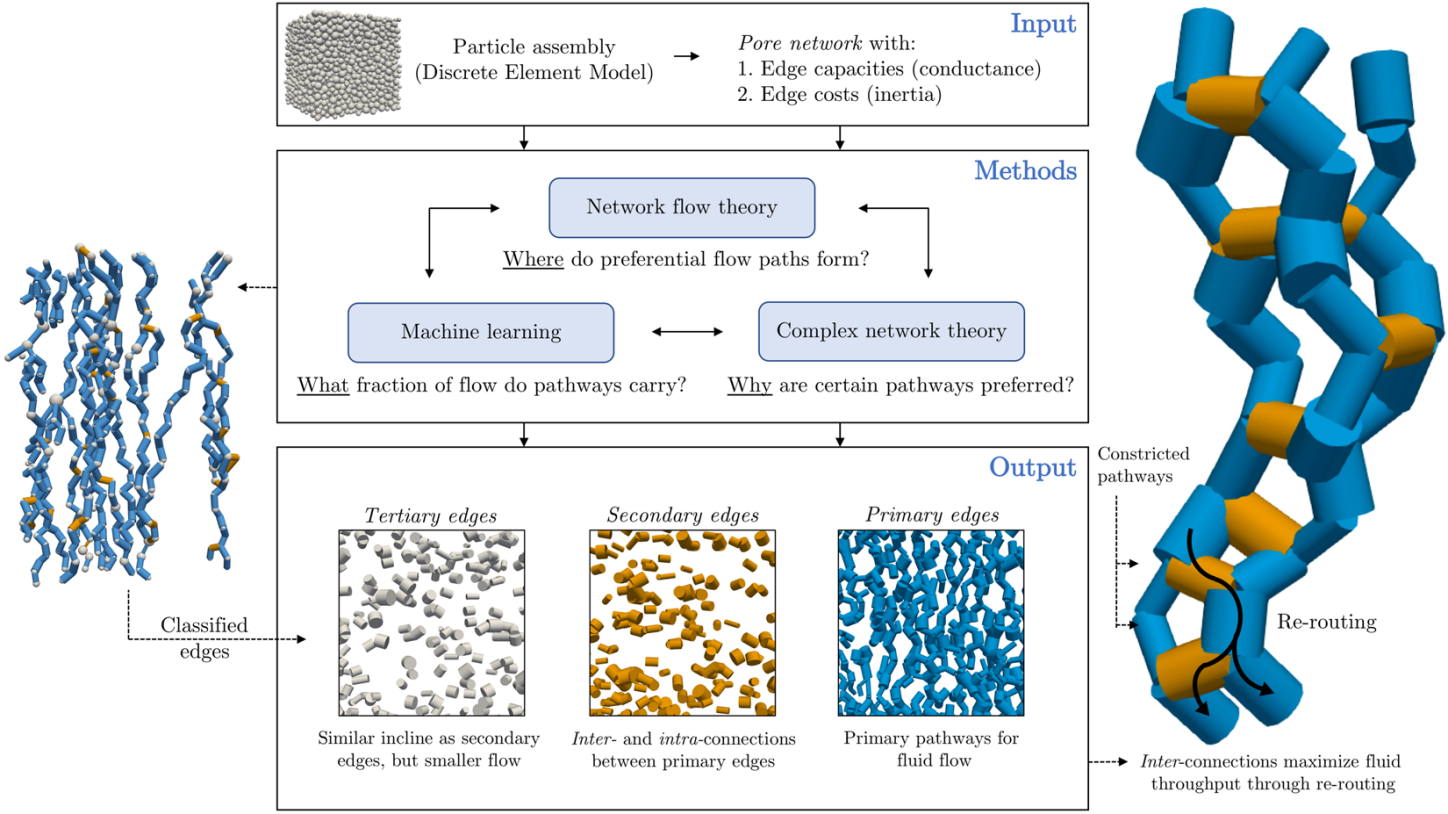
Optimal pathways through a failing granular material give rise to a hierarchy of primary, preferential flow pathways, laterally supported by secondary, bridging pathways. Snapshots of an assembly of spherical particles subjected to a triaxial test are extracted from the discrete element simulation (Input). A pore network is constructed for each assembly, in which nodes represent pores and edges represent throats (Input). Next, the Maximum Flow, Minimum Cost (MFMC) algorithm is applied to the pore network to extract a sub-network *P* of optimal flow paths. Using a logistic regression classifier, we distinguish between edges in P that can be correctly predicted as being part of *P* (primary edges, blue), incorrectly predicted as not being part of *P* (secondary edges, orange) and the remainder (tertiary edges, grey). Further examination of these edges using complex network theory reveals secondary edges act either as intraconnecting pathways, embedded in chains of primary edges, or as interconnections, bridging the primary pathways and re-routing flow to maximize throughput, as shown on the right-hand side.

Figure 1 provides a summary of the data generated, methodology used and our key results, further expanded on in this section.

### Experiment

**Figure 2:**
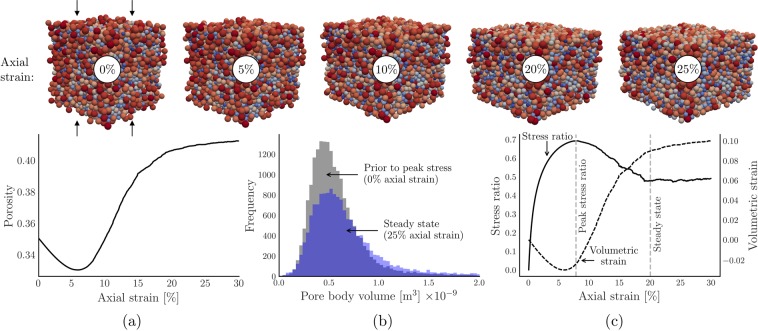
A previously published and validated discrete element simulation is used to model a deforming granular material. Summarized results for the discrete element simulation. Showing (**a**) porosity versus axial strain, (**b**) pore body volume distributions, prior to peak stress and in the steady state, and (**c**) the evolution of the stress ratio and volumetric strain.

To capture the evolution of the pore space in a deforming granular material in the presence of dilatant shear zones, we subject a dry assembly of 6,776 polydisperse spheres to a triaxial shear test. This sample is one of a series of shear tests which has been comprehensively examined and reported elsewhere^10,48,49,51^ (see also Ord *et al*.^52,53^ for similar tests). The mechanical response of the sample is summarized in Fig. 2. During the test, the assembly expands globally in the horizontal directions under a relatively high constant confining pressure and a constant vertical axial strain rate, as evidenced by the increase in porosity and volumetric strain. Pore bodies increase in size accordingly, as shown in Fig. 2(b), merging smaller pores (decreasing their frequency) and developing a long tail in the distribution. Peak stress ratio occurs at about 7% axial strain and the assembly is in steady state at 20% and beyond. Snapshots of the assembly at 430 regularly-spaced strain intervals between 0 and 30% axial strain are the main input data for the remainder of the analysis.

### Maximum flow, minimum cost

**Figure 3:**
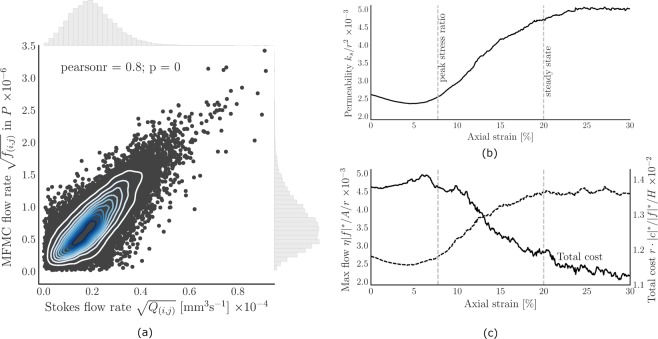
Micro-scale and macro-scale output of the network flow model, in the form of the edge flows, maximum flow and associated cost of transmission shows good agreement with well-known trends in stress ratio, volumetric strain, Stokes flow and permeability for deforming granular materials. (**a**) Correlation between (square-root transformed) network model flow rates and Stokes flow rates at the edge level for 20% axial strain, including marginal distributions and density contours to indicate the joint distribution. (**b**) Evolution of the (dimensionless) permeability, normalized by the squared particle radius *r*^2^, plotted against axial strain as the sample fails. (**c**) Dimensionless maximum flow |*f*|^*^ and total cost |*c*|^*^, as computed using the Maximum Flow, Minimum Cost algorithm. The dimensionless maximum flow is calculated by multiplying with the dynamic viscosity *η* and dividing by the cross-sectional area *A* and mean particle radius *r*. Total cost is normalized by the mean particle radius *r*, maximum flow |*f*|^*^ and height *H* of the assembly.

A pore network is constructed independently for each snapshot of the deforming assembly, embedding local connectivity of the pore space by modeling pores as nodes and throats as edges in a graph. This particular pore network construction approach has been previously validated in^54,55^. Each pore network is the input for the Maximum Flow, Minimum Cost (MFMC) algorithm. MFMC aims to identify the optimal flow pathways through the pore network, starting at a collection of defined inlet nodes at the top of the assembly (shown in Fig. 1) and ending at the outlet nodes at the bottom. Optimality in this network flow model is achieved by maximizing *flow* (subject to the *capacity*) while minimizing *cost*. To mimic fluid flow, we define capacity as the fluid conductance of a simplified cylindrical throat, calculated using the Hagen-Poiseuille law. Under the corresponding assumptions of tube flow, wide throats that connect large pores over a short distance have large capacity. Cost is defined as an inertia penalty value. If an edge (*i*, *j*) is poorly aligned with other edges connected to nodes *i* and *j*, then the cost value is high. This penalty models the resistance of fluid to flow ‘around a bend’, due to inertia of the fluid. With capacity defined as conductance and cost representing inertia effects, we essentially provide the network flow model with some of the key ingredients of the Navier-Stokes equation for viscous fluid flow. Defining capacity as conductance also allows us to conveniently (and efficiently) compute the approximate numerical permeability. Assuming saturation, we solve the Stokes equation for each pore network, imposing a pressure difference of l Pa over the inlet- and outlet nodes and inducing local hydraulic gradients in the order of 10 Pa/m. From the resulting pressures and flow rates, the permeability is derived using Darcy’s law. The result is included in Fig. 3(b) and shows that the porosity in Fig. 2(a) and volumetric strain (capturing dilation) in Fig. 2(c) follow the same trend as permeability, as expected. The permeability increases by approximately a factor two between the minimum at 5% axial strain and the maximum in the steady state.

Given the edge capacities and costs, the MFMC algorithm returns a sub-graph (denoted *P* and referred to as the *preferential flow network*) of nodes and edges that make up the (optimal) *preferential flow pathways* through the pore network. The percolating network *P* transmits the maximum flow from inlet nodes to outlet nodes, where the maximum flow is the maximum number of units of flow that can be ‘pushed’ through the network, bounded by edge capacities. Due to the absence of any global physical bottlenecks, the majority (between 72% and 77%) of edges in the original pore networks at different strain intervals are part of the corresponding MFMC sub-graph *P*. A small excerpt of *P* is shown on the right-hand side of Fig. 1. The maximum flow and corresponding total (summed) cost of transporting the maximum flow is plotted in Fig. 3. Maximum flow follows approximately the same trend as permeability, suggesting good resemblance between *macro-scale* MFMC and Stokes flow behavior. In the Supplementary Material, the Stokes flow permeability is shown to correspond well to the permeability derived from the Navier-Stokes equation for fully saturated sphere packing geometries. The Navier-Stokes permeability has previously been validated against experimental values^56^, suggesting indirectly that MFMC can capture realistic physical fluid flow behavior. Total cost follows the inverse trend of permeability, capturing the intuition of increased permeability in the presence of ‘cheaper’ flow pathways. At the micro (edge) scale, shown in Fig. 3(a), MFMC flow is well-correlated with Stokes flow rates, with a Pearson correlation coefficient of 0.8 at 20% strain. The (statistically significant) correlation coefficient otherwise varies between 0.72 at 0% strain and 0.81 in the steady state (*p* < 2 · 10^−16^).

### Primary, secondary and tertiary flow

Having defined our network flow model, and having validated the output of the algorithm by comparing flow at the micro-structural and macro-structural level, we focus on *why* preferential flow occurs in certain edges next. To do so, we define a binary classifier to predict inclusion or exclusion *χ*_(*i*,j)_ of a particular edge in the MFMC sub-graph *P*, i.e. *χ*_(*i*,j)_ = 1 if (*i*, *j*) ∈ *P* and *χ*_(*i*,j)_ = 0 otherwise. For the results in Table 1, the classifier model is fitted on a randomly selected 80% of edges in the sample with axial strain 21.54% (21737 edges). Generalization performance is obtained by testing the fitted model on the remaining 20% of edges, for which we obtain an area under the *ROC* curve equal to 0.76 (0.5 corresponds to random guessing, while 1.0 would imply a perfect prediction). For other strain rates (repeating the model fitting process described here), the area under the ROC curve is approximately similar, varying between 0.75 and 0.81.

**Table 1:** Features for each edge (*i*, *j*) used in the logistic regression model. Notation of features and coefficients refers to Eq. (7).

Feature name	Equation	Significance	Odds ratio	95% conf. interval
Edge cost	*X*_1_ = *c*_(*i*,*j*)_ in Eq. (4)	*p* < 2 · 10^−16^	${e}^{{\beta }_{1}}=0.55$	[0.53, 0.57]
Throat area	*X*_2_ = *A*_(*i*,*j*)_	*p* < 2 · 10^−16^	${e}^{{\beta }_{2}}\mathrm{=0.84}$	[0.81, 0.87]
Adjacent pore volume	${X}_{3}={\bar{V}}_{(i,j)}$ in Eq. (1)	*p* < 2 · 10^−16^	${e}^{{\beta }_{3}}\mathrm{=1.36}$	[1.30, 1.42]
Incline	*X*_4_ = *θ*_(*i*,*j*)_ in Eq. (2)	*p* < 2 · 10^−16^	${e}^{{\beta }_{4}}\mathrm{=2.59}$	[2.48, 2.69]
(Constant)		*p* < 2 · 10^−16^	${e}^{{\beta }_{0}}\mathrm{=3.66}$	[3.52, 3.80]

The odds ratio of the cost value *c*_(*i*,*j*)_ (0.55) in Table 1 is smaller than one, indicating that every unit increase in the standardized value of *c*_(*i*,*j*)_ decreases the odds of *χ*_(*i*,*j*)_ = 1 by a factor 0.55. The odds ratio for throat area is closer to one, suggesting that a larger throat area has a slightly negative impact on the odds of an edge being included in *P*. For ${\bar{V}}_{(i,j)}$ and *θ*_(*i*,*j*)_, we observe ${e}^{{\beta }_{4}} > {e}^{{\beta }_{3}}\mathrm{ > 1}$, which implies that these features positively impact the odds of being included in *P*. A unit increase in the standardized incline, in particular, increases the odds by a factor 2.59, capturing the intuition that edges better aligned with the vertical pressure gradient of the assembly are more likely to be part of the preferential flow network. In contrast with throat area, pore volume also has a positive effect on the odds. For a relatively narrow throat size distribution and in the absence of major bottlenecks, we attribute this difference to the preference of flow to find the most direct routes across the sample, as provided by pores of larger size and edges aligned with the global pressure gradient.

**Figure 4:**
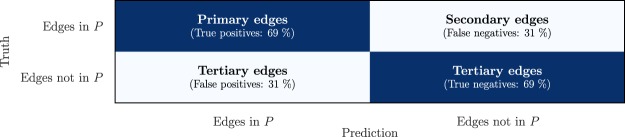
Confusion matrix for the prediction on the assembly with axial strain 22.94%.

Having fitted the logistic regression model, and having tested the ability of the model to generalize, we re-fit the model to 100% of edges in the the assembly with axial strain 21.54% and predict inclusion or exclusion in *P* for each edge in the pore network at the next strain state (21.61%). The confusion matrix for this result is shown in Fig. 4.

For this sample, 69% of edges in *P* are correctly predicted as *χ*_(*i*,*j*)_ = 1 (true positives). The remaining 31% are incorrectly predicted as *χ*_(*i*,*j*)_ = 0 (false negatives). The difference between true positives and false negatives gives rise to a distinction between edges in *P* that can be predicted with physical features and a binary classifier, versus edges in *P* that are less predictable. As such, we designate edges in *P* that are correctly predicted as *χ*_(*i*,*j*)_ = 1 as *primary edges*. Edges that are part of *P* but incorrectly predicted as *χ*_(*i*,*j*)_ = 0 are called *secondary edges*. All edges not in *P* are referred to as *tertiary edges*. Figure 1 shows an example of this hierarchy. For other strain rates (repeating the model fitting process as described), the true positive rate and true negative rate are approximately similar to the rates shown in Fig. 4, varying between 68% and 75%.

**Figure 5:**
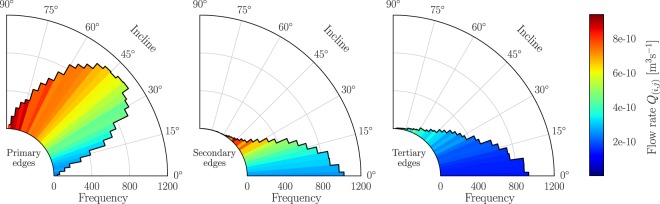
Secondary pathways are poorly aligned with the pressure gradient, yet exhibit favorable flow rates, suggesting an important role in bridging preferential flow pathways. Radial distribution of the incline of primary edges (left), secondary edges (middle) and tertiary edges (right). The color of each bar indicates the average flow rate of all edges in the corresponding range of inclines.

To assess this distinction further, the distribution of the incline of primary, secondary and tertiary flow edges is shown in Fig. 5 along with the Stokes flow rates in corresponding edges.

Clearly, edges closer aligned with the pressure gradient (at 90°) have higher flow rates, as expected. The majority of edges are primary, with an incline centered around approximately 45° and exhibiting high flow rates (*Q*(_*i,j*_) = 5.9 · 10^−10^ m^3^ s^−1^, on average). In this sense, primary edges bear resemblance to the preferential ‘hotspots’ of fluid flow, as described in the introduction^7,8^. Tertiary edges, in contrast, are more horizontally aligned (21°, on average), perpendicular to the pressure gradient, and have a considerably lower average flow rate (2.1 · 10^−10^ m^3^ s^−1^,). Interestingly, secondary edges have even smaller inclines (15°, on average), yet the flow rates are considerably higher (4.0 · 10^−10^ m^3^ s^−1^, on average) than the flow rates of tertiary edges. Indeed, comparing the square-root transformed flow rate for primary, secondary and tertiary edges in a one-way ANOVA test rejects the null hypothesis that the means are the same (*F*(2,33963) = 3365, *p* < 2 · 10^−16^). A post-hoc Tukey test also confirms that there is a statistically significant difference between each of the three pairs of means (*p* < 2 · 10^−16^). In contrast, the average pore volume of adjacent nodes is 1.36 · 10^−9^ for secondary edges and 1.40 · 10^−9^ for tertiary edges, which is not a statistically significant difference (Tukey test, *p* = 0.62, using square-root transformation). In conclusion, secondary edges have a less favorable incline than tertiary edges, and similar adjacent pore volume, yet the flow rates for secondary edges are significantly higher. These conclusions also hold for flow networks at other axial strain states, including pre-peak stress.

These observations suggest that secondary edges play a distinct role in the transmission of fluid through the pore space. Despite less favorable circumstances in terms of incline, secondary edges have significantly higher flow rates than tertiary edges. Further qualitative examination of these pathways, shown on the right-hand side of Fig. 1, reveals that secondary edges are either *intra*connections or *inter*connections for the preferential pathways of primary edges. Intraconnections are embedded in chains of primary edges, forming preferential flow pathways that span the assembly from inlets to outlets and carry the fluid across. Interconnections, in contrast, *bridge* these preferential pathways, providing the ability to re-route flow in order to maximize the overall throughput. Interconnecting secondary edges therefore play a crucial role in the global optimization of finding pathways that maximize flow while minimizing cost.

### Impact of pathway hierarchy on permeability

**Figure 6:**
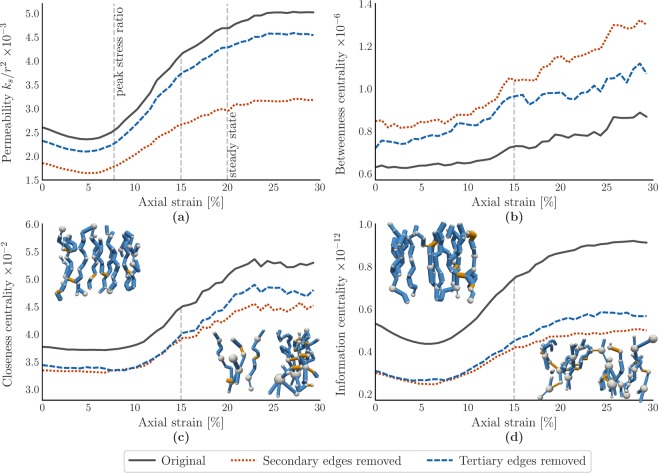
Removing secondary, bridging pathways has a stronger negative effect on permeability and centrality than removing tertiary pathways. Moreover, secondary pathways become more important for the overall transmission beyond peak stress and into the steady state. Impact of removing secondary or tertiary edges on (**a**) permeability, (**b**) edge-averaged betweenness centrality for all shortest paths between inlets and outlets, (**c**) node-averaged closeness centrality for all shortest paths, (**d**) node-averaged information centrality. Insets show pre- and post-peak stress excerpts of the 10% lowest-cost pathways in *P*, with nodes and edges sized by pore size and throat size, respectively. Primary edges are colored blue and secondary edges are colored orange.

The importance of inter- and intra-connecting flow is further highlighted by removing all secondary edges from the original pore network and re-calculating the permeability shown in Fig. 3. To do so, we repeat the process described in the previous section, fitting a logistic regression classifier for every assembly in the loading history and identifying primary, secondary and tertiary edges. In Fig. 6(a), the impact on permeability by removing secondary edges is compared to the impact on the permeability when tertiary edges are removed instead. Removing primary edges disconnects the inlets from the outlets altogether, hence a permeability for this type of removal is not shown.

We observe a pronounced difference of the impact on permeability if secondary edges are removed, compared to removing tertiary edges. On average, permeability is reduced by approximately 10% throughout the loading history if tertiary edges are removed. Prior to peak stress, the reduction is 30% for secondary edges, increasing to a 37% reduction in the steady state. It should be noted that the number of secondary edges is generally slightly smaller than the number of tertiary edges in each assembly, with an average of 7914 and 8929 edges across all strain states, respectively. The per-edge percentual reduction in permeability is still consistently and considerably larger for secondary edges, however: −33.6%/7914 = −4.2 · 10^−3^% for secondary edges, versus −9.9%/8929 = −1.1 · 10^−3^% for tertiary edges.

### Impact of pathway hierarchy on transmission efficiency

We quantify the association between permeability, preferential flow, the primary-secondary-tertiary distinction and network connectivity patterns using measures of complex network centrality. We calculate the average *closeness centrality*, *betweenness centrality* and *information centrality*. Closeness and betweenness are based on shortest paths, assuming that flow occurs only along these shortest paths and does not split. More specifically, an unweighted shortest path between node *i* and *j* is the route through the pore network that requires the minimum number of edges. The closeness centrality of a node *i*, in turn, is the reciprocal of the summed shortest path lengths to every other node in the pore network. A larger average closeness centrality suggests that most nodes in the network are relatively well-connected to all other nodes. Edge betweenness centrality is defined as the fraction of all shortest paths between pairs of nodes in the pore network that pass through the edge under consideration. In this case, only the paths that connect an inlet node with an outlet node are considered. Large average betweenness centrality indicates that shortest paths through the pore network are relatively localized and less spread out. Information centrality models connectivity through electrical current flow paths that can split and may follow any (potentially sub-optimal) route. This type of centrality is calculated for a node *i* as the reciprocal of the summed potential differences between *i* and every other node *j*. The edge resistance, a required input of the algorithm, is conveniently calculated as the inverse of the conductance capacity used in our network flow model. High average information centrality, in our case, suggests a more well-connected network in terms of *all* possible paths between nodes.

Figure 6(b–d) illustrates the impact of removing all secondary or all tertiary edges from the pore network. Betweenness centrality is edge-averaged, while closeness- and information centrality are averaged across all nodes.

The betweenness centrality results in Fig. 6(b) show that shortest paths between inlets and outlets gradually become more localized following peak-stress. More interestingly, shortest paths between inlets and outlets become considerably more localized when secondary edges are removed, compared to removing tertiary edges, up to a factor two at 30% axial strain. The removal of inter-connecting edges would contribute to such localization, eliminating the capacity to re-route and focusing shortest paths down the remaining routes connecting inlets with outlets. The difference in closeness centrality by removing secondary and tertiary edges in Fig. 6(c) is negligible until 15% axial strain. Beyond 15%, removing secondary edges decreases closeness centrality more considerably. The transition suggests that inter- and intra-connections, as provided by secondary edges, become more important in terms of maintaining connectivity as the sample fails. Indeed, the impact on betweenness centrality in Fig. 6(b) of removing secondary edges is also more pronounced in the steady state, compared to prior axial strain values.

Lastly, the trend in information centrality (Fig. 6(d)) is remarkably similar to the trend in permeability. This may not be entirely surprising, given the use of conductance in both results, and the corresponding similarities between calculating pressure differences using Stokes’ law and electrical potential differences using Kirchhoff’s laws. The Pearson correlation coefficient between the permeability and closeness centrality is equal to 0.975 (*p* < 2 · 10^−16^), compared to 0.995 (*p* < 2 · 10^−16^) for the correlation between permeability and information centrality. Note that the decrease in closeness centrality when secondary/tertiary edges are removed is smaller than the decrease in information centrality. We attribute this difference to the fact that information centrality counts ‘distance’ as an aggregated amount of information flow along *all possible paths*. Closeness centrality only considers the shortest path. As a result, removing edges is more impactful for information centrality. The impact of removing secondary edges in terms of information centrality is more pronounced after 15% axial strain. The decrease in shortest-path connectivity (Fig. 6(c)) and connectivity of all paths (Fig. 6(d)) through removal of secondary edges may contribute to the 37% permeability reduction (Fig. 6(a)) in the steady state, up from 30% prior to peak stress.

### Impact of deformation on pathway hierarchy

**Figure 7:**
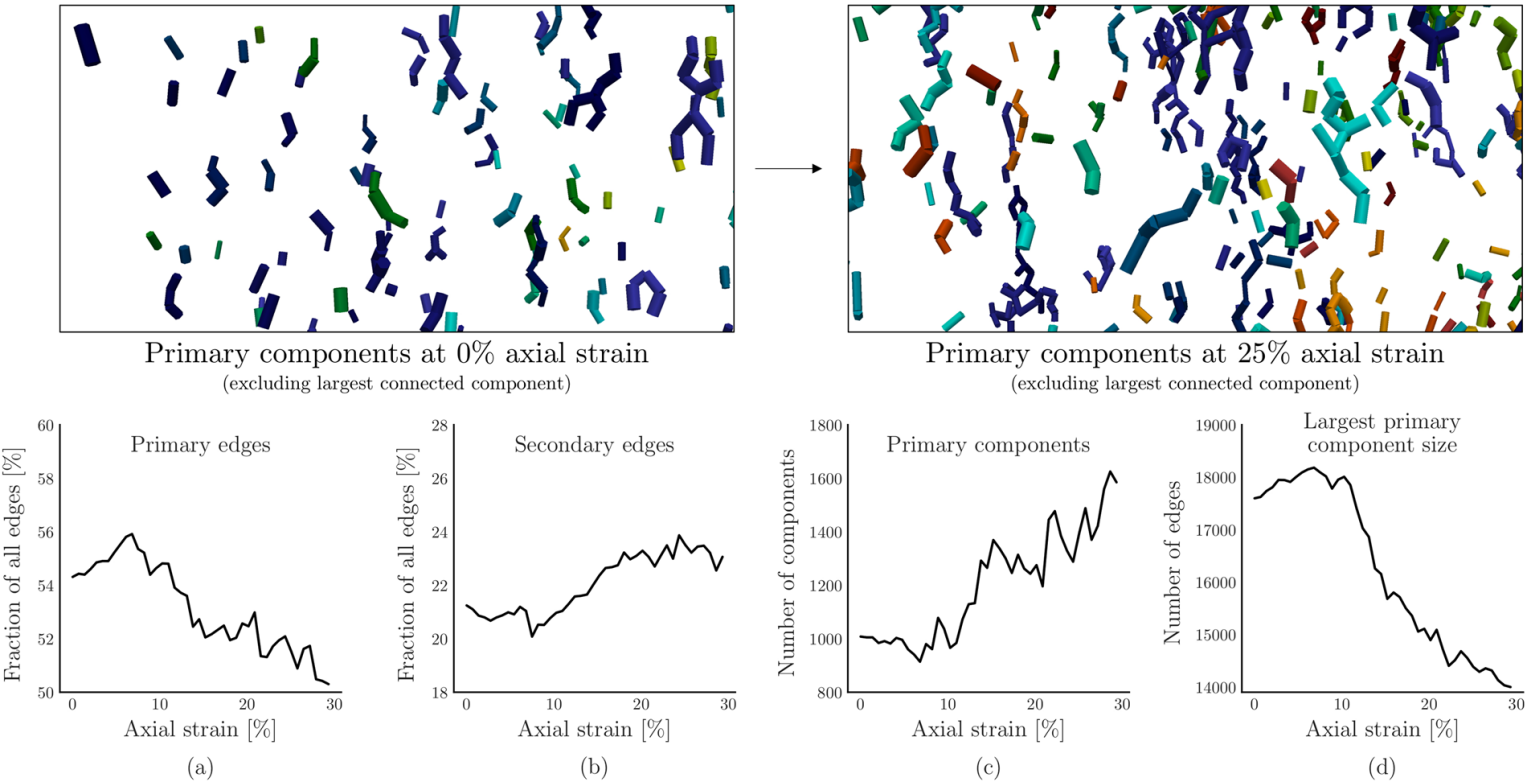
A decrease in the fraction of primary edges coincides with an increase in the number of primary components, indicating an increasingly fragmented primary flow network in the steady state. Showing (**a**) fraction of primary edges, (**b**) fraction of secondary edges, (**c**) number of primary components, and (**d**) number of secondary components. Insets at the top illustrate the primary components at 0% and 25% axial strain.

Finally, we employ the proposed definition of preferential flow and the resulting hierarchy of primary-secondary-tertiary flow pathways to shed light on the impact of deformation on preferential flow pathway connectivity and structure. First, we investigate the frequency of primary and secondary edges, as well as the structures that they form. To this end, we introduce the notion of *primary components* as the disconnected components in *P* if all but the primary edges are removed. Similarly, the *secondary* components are the disconnected components in *P* if only secondary edges are retained. Figure 7 compares the frequency of primary and secondary components with the relative frequency of the primary and secondary edges. Figure 7(a,b) show that a decrease in the percentage of primary edges coincides with a slight increase in the relative frequency of secondary edges. The largest connected component, quantified in size in Fig. 7(d), connects 80–90% of the primary edges at 0% axial strain, in addition to the approximately 1000 other primary components shown Fig. 7(c). The subsequent decrease in size of the largest component coincides with an increase in the number of primary components. From this, we derive that the primary flow structures become increasingly fragmented as the assembly reaches the steady state. Given the favorable flow characteristics of primary edges, shown in Fig. 5, this fragmentation would have a negative impact on permeability. Yet the permeability increases with increasing axial strain, as shown in Fig. 3(b). We attribute this counter-intuitive behavior to the increasingly prominent role of secondary edges. The frequency of these inter- and intra-connecting edges does increase slightly, as shown in Fig. 7(b). More importantly, the results in Fig. 7 provide further evidence for the idea that secondary edges provide re-routing capabilities for the (increasingly fragmented) primary components, as the sample deforms, corroborating similar observations in the centrality measures presented in Fig. 6.

**Figure 8:**
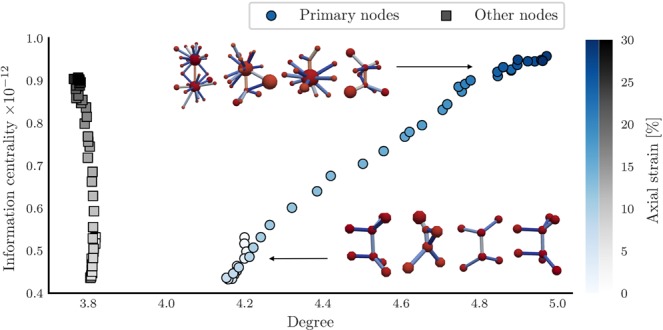
During deformation, and particularly in the steady state, nodes in primary pathways of flow become more connected to other nodes, both locally, in terms of degree, and across multiple scales, in terms of information centrality. Non-primary nodes also gain in multi-scale connectivity, but do not become more locally connected. Feature vector trajectory, in terms of degree and information centrality, for primary and non-primary nodes. Color gradient indicates the axial strain. Insets illustrate nodes with some of the highest centrality values prior to peak stress, and in the steady state.

Figure 8 compares the evolution of local connectivity in terms of degree (i.e. pore coordination number), with changes of multi-scale connectivity in terms of information centrality, as the axial strain increases. Each point represents an average across all nodes in the stated category. A node is considered primary if its primary degree (number of adjacent primary edges) is larger than the global average primary degree across all nodes. Generally, the ratio of primary nodes to other nodes is approximately 1:2.

For primary nodes, local connectivity increases as the axial strain increases, from an average of 4.2 connected edges prior to peak stress to an average degree of 5 in the steady state. The average degree of non-primary nodes, in contrast, remains constant at 3.8 edges, despite continued physical deformation of the material. Both primary and non-primary nodes become more connected to all other nodes in terms of information centrality post peak stress, with primary nodes achieving slightly higher values towards the end of the shear test. Closer inspection of the associated network structures (insets in Fig. 8) reveals that primary nodes post peak stress, and into the steady state, become highly-connected, micro-structural information (i.e. fluid) hubs. This connectivity may be associated with the above-average flow rates for primary edges (connected to these primary nodes) and below-average flow rates for tertiary edges in Fig. 5. The trend in degree for primary nodes also suggests a correlation with permeability, which is found to be equal to 0.982 (*p* < 2 · 10^−16^) in terms of the Pearson correlation coefficient, similar to the correlation between permeability and information/closeness centrality.

## Discussion

In this section, we connect our insights with existing definitions of preferential flow and prior experimental work. The proposed method to identify preferential flow pathways and corresponding functional groups extends existing interpretations in the following ways. As outlined in the introduction, preferential flow has previously been defined by the delineation of the liquid phase in relatively mobile and immobile parts^1^, where the preferential (mobile) part bypasses a fraction of the porous matrix^2^, through large inter-aggregate voids also known as macropores^3^. Indeed, in Fig. 3(a), we observe good correlation between what the network flow model predicts to be the fast-flowing, high-throughput fraction of the pore network, and what Stokes flow predicts as such. As a result, the edge flow *f*_(*i*,*j*)_ provided by MFMC may be interpreted as micro-structural quantification of confidence that the edge is preferred, in addition to the macro-structural confidence parameters provided in Table 1). We also observe a statistically significant difference in the average flow rate through primary edges (*Q*_(*i*,*j*)_ = 5.9 · 10^−10^ m^3^ s^−1^) and tertiary edges (*Q*_(*i*,*j*)_ = 2.1 · 10^−10^ m^3^ s^−1^) in Fig. 5. The secondary edges exhibit an intermediate average flow rate of 4.0 · 10^−10^ m^3^ s^−1^, however, suggesting an extension of the mobile-immobile distinction to include a third part for the bridging pathways that provide re-routing capabilities for the primary preferential pathways and maximize overall throughput, as hypothesized in the introduction. Secondary interconnections can be observed in the experimentally observed preferential fluid flow pathway images of Ju *et al*.^12^, though they are not fully captured by the shortest path model presented therein. Shortest paths alone do not account for pathway capacity and the corresponding inherent interplay between redundancy and rerouting in complex systems. As a result, the secondary (bridge-like) connections between primary preferential flow pathways observed in X-ray CT scans of soil columns^19^ cannot be captured using shortest paths alone. Addressing this issue, MFMC takes both cost (shortest paths) and capacity into account, from which the distinction between primary and secondary (bridging) preferential route segments emerges (e.g. see Fig. 1). In capturing primary, secondary and tertiary pathways of preferential fluid flow, our MFMC results corroborate the self-organizing, complex linkage continuum observed by Sidle *et al*.^16^ and Luo *et al*.^19^.

Our definition of preferential flow also corroborates the well-known preference for larger (macro)pore sizes^3^, as indicated by the positive odds (1.36) of the pore volume term in the logistic regression model (Table 1). Rather than capturing preferential flow using individual parameters such as (macro)pore size, our model considers a multitude of multiscale factors influencing preferential flow simultaneously (pore size^3^, throat area^12^, path alignment with the pressure gradient and surrounding pathways^16,19^) to obtain the optimal decision boundary between primary and secondary pathways. As such, the classifier provides a direct statistical interpretation for other relevant factors as well: the odds of an edge being included in the preferential flow network decrease by a factor 0.55 for every unit increase in edge cost, for example, and increase by a factor 2.59 for every unit increase in edge incline. Interpreted as such, the logistic regression model provides deeper insights into why certain parts of the porous medium are predicted as being part of the preferential flow network by our network flow model.

In the observations of inter- and intra-connecting edges summarized in Fig. 1, a particular analogy emerges with the two-tier hierarchical architecture observed in contact networks and force chains. Prior to peak stress, the sample is compressed along the vertical axis. The material can sustain the applied load through the formation of what is known as the *strong*, load-bearing force network along particle contacts, laterally supported and bridged by contacts in the *weak* force network^38,57^. A loss of lateral support and the phenomenon of *buckling force chains* results in subsequent failure of the assembly post peak-stress^51^. In rock formations, such events are associated with the formation of fracture patterns^53^. Figure 3(b) and 3 show that permeability, maximum flow and total cost are approximately constant in the critical regime, aside from minor fluctuations associated with local compression and dilation resulting from the spatiotemporal formation and buckling of force chains supporting the material. The plateauing behavior of the maximum flow in the steady state has also been observed in force transmission studies using MFMC^36^. In the same way that force is distributed under loading by forming a hierarchical support structure, our observations suggest that preferential fluid flow maximizes throughput by bridging primary flow pathways with secondary, horizontally-oriented connections. As such, juxtaposing our work with the fracture pattern model of Ord and Hobbs^53^, we observe that preferential flow is routed not just between macropores *within* regions of dilation (i.e. fracture patterns), but also, through secondary (multiscale) connections, *between* such regions. The supporting structures for force transmission and preferential flow are also inherently connected: compression and dilation due to the formation and buckling of force chains continuously alter the pore space^10,37^. Indeed, Fig. 6 shows that the secondary connections between primary flow pathways become increasingly important for preferential flow as the assembly dilates beyond peak-stress, furthering the idea of Allaire *et al*.^4^ that lateral flow connections are at least as important as preferential flow in the direction of gravity (for overall throughput). Additionally, the loss of inter- and intra-connecting edges analyzed in Fig. 6 demonstrates that secondary pathways are responsible for a larger fraction of the connectivity and physical flow than tertiary edges.

Related work has shown that closeness centrality is more strongly correlated with permeability than most other complex network parameters, including betweenness centrality^54,55^. Building on this outcome, the results in Figs. 6 and 8 suggest that information centrality, previously not considered due to computational costs, is (linearly) more strongly correlated with permeability than closeness centrality. We noted in the discussion of Fig. 8 that permeability is also significantly correlated with the average degree of primary nodes, yet not correlated with the average degree of non-primary nodes. While we cannot prove causality, these results suggest that the local increase in connectivity of primary nodes (depicted in the insets of Fig. 8) may be responsible for the increase in multi-scale connectivity (closeness centrality and information centrality) and permeability post peak stress. Translating these insights back to the physical domain, increased local connectivity of primary nodes implies that pores likely to be part of preferential flow pathways become (on average) more connected to neighboring pores during deformation of the material. The degree of pores outside the preferential flowpaths, in contrast, does not increase. Further inspection shows that primary nodes are likely to represent larger pores, as predicted by the logistic regression model (${e}^{{\beta }_{3}}=1.36$ in Table 1) and consistent with the macropore definition of preferential flow^3^. This insight is also consistent with observations by Ju *et al*.^12^, who observed a preferential flow path preference for macropores both in experimental measurements and their shortest path model. Large pores, in turn, are the result of local dilation and shear bands that follow the aformentioned buckling force chains post peak-stress^37,51,53^. Additionally, we observe in Fig. 6 that the negative impact on permeability and connectivity by removing secondary edges (bridging primary nodes/edges) is most pronounced in the steady state. We conclude that preferential flow benefits from the onset of failure at peak-stress, a result that was previously hypothesized in 2D by Russel *et al*.^10^. While primary, flow-promoting edges decrease in number and become increasingly fragmented post peak-stress, Fig. 7 shows that secondary edges compensate for this by providing the inter- and intra-connecting routes that maximize the throughput, ultimately resulting in an increase in permeability.

This work is a first step in defining and predicting preferential flow structure for more complex materials using MFMC. For saturated media, as considered in this work, comparing MFMC to Navier-Stokes fluid flow^56^ and considering coupled interactions (e.g. pore pressure, internal erosion and fault slip^15^) could provide further insights into the applicability and limitations of MFMC, and the structure of preferential fluid flow pathways. Particularly in the presence of higher Reynolds’ numbers and stronger inertia effects, the contribution and role of secondary edges may become more or less pronounced. Beyond the assumption of full saturation, hydrological effects in unsaturated media, typically modelled by Richards equation^23^, should also be investigated, including wettability effects, drainage and air entrapment^19^. In our idealized particle assemblies, the network flow model highlights non-linear interdependencies between microstructure and preferential path formation in fully saturated granular media. For real granular materials, such non-linear and non-intuitive responses will likely be even more pronounced. A comparison to real fluid flow obtained from MRI scans or X-ray tomography^50^, similar to the work by Ju *et al*.^12^ for shortest paths, could provide experimental (network and capacity) input and validation for MFMC. The MFMC framework is exemplified herein with idealized realistic virtual samples and its resulting insights are contrasted with relevant observations available in literature. Beyond structure, applying MFMC to real pore geometries may also provide further insights into the physical dimensions of preferential flow pathways. As long as a pore network representation with corresponding capacities is available, MFMC is generally applicable to void descriptions in permeable media.

## Methods

### Discrete element simulation

The discrete element model used here is from Pucilowski *et al*.^48,49^. It is a three-dimensional analogue of a well-studied in-plane biaxial compression test^51^ under a relatively high constant confining pressure to induce dilation during shear. Indeed, these samples were designed for characterization of dynamics of transmission in a densely-packed 3D granular sample up to and during failure when the sample deforms in the presence of fully developed dilatant shear zones. As shown by Ord^52^, fluid flow is enabled by regions of dilatancy (e.g. shear zones). The model parameters are summarized in Table 2. The height coordinate is *z* and the width and depth coordinates are *x* and *y*, respectively. In summary, a normally consolidated drained triaxial test is simulated for a dry, sand-like specimen. As is common in discrete element methods, a friction-free approach is used to simulate air pluviation. 6,776 spherical particles are placed in a friction-free, stress-free, non-contacting, and gas-like state in a cubic box without gravity. The walls are subsequently moved inwards to compress the sample to 495 kPa constant confining pressure. When the assembly reaches a static equilibrium, particle velocities are reset and particles are assigned the friction coefficients listed in Table 2. The horizontal (top and bottom) walls are assigned the same sliding friction coefficient as the particles (0.7), while vertical walls in the remaining simulation are still assumed to be frictionless. This way, no additional artificial shear is imposed to the virtual specimen. Next, the assembly is subjected to a triaxial shear test, with a constant axial strain rate of −0.05 s^−1^ in the vertical direction. During the test, the assembly expands globally in horizontal directions under constant confining pressure. The state of the dry assembly is extracted at 430 equally spaced iterations, corresponding to 430 stages of axial strain between 0 and 30%, for further analysis.

**Table 2:** Simulation parameters used in DEM.

Number of particles	6,776
Particle radii (from uniform distribution)	(0.76 − 1.52) × 10^−3^ m
Density	2650 kg/m^3^
Young’s modulus	9.19 × 10^7^ Pa
Poisson’s ratio	0.5
Rolling stiffness coefficient^37^	1.0
Rolling friction coefficient	0.7
Sliding friction coefficient	0.02
Strain rate	−0.05 *s*^−1^
Constant confining pressure (in *x* and *z* direction)	4.95 × 10^5^ Pa
Initial void ratio	0.5
Initial dimensions (*x* × *y* × *z*)	109.23 × 109.20 × 109.26 10^−3^ m
Final dimensions (*x* × *y* × *z*)	133.25 × 132.09 × 80.97 10^−3^ m

### Pore network construction

We use the modified Delaunay tessellation (MDT) approach to construct the pore networks, originally proposed by Al-Raoush *et al*.^58^, with the adaptations introduced by van der Linden *et al*.^54^. For a detailed description of the algorithm and a comparison with the original MDT approach, refer to^55^. The Delaunay tessellation delineates the pore space using tetrahedra, for which the vertices coincide with the particle centroids. The tetrahedra are assumed to encapsulate pores, while throats are assumed to coincide with the tetrahedral faces. Al-Raoush *et al*.^58^ recognized that the tessellation may unnecessarily sub-divide pores, however, and introduced the idea of merging tetrahedra to remedy this issue. We merge a pair of tetrahedra if the areal porosity on the shared face exceeds a pre-defined threshold^54^. The threshold is fixed at 0.4, which has been shown to achieve a good balance between under- and over-merging^55^. Following the merging procedure, network nodes are assigned to individual or merged collections of tetrahedra, connected by an edge if the tetrahedra share a face. Nodes are labelled as inlet (outlet) nodes if one or more tetrahedra contains a face exposed to the top inlet (bottom outlet) plane.

Pore volume *V*_*i*_ for node *i* is calculated by summing the void volume encapsulated by the corresponding tetrahedron or tetrahedra. Similarly, throat area *A*_(*i*,*j*)_ is obtained by summing the void area on all shared faces. The derived variables of adjacent average pore volume and edge incline in Table 1 are defined as:

1 $${\bar{V}}_{(i,j)}=({V}_{i}+{V}_{j})/2$$

2 $${\theta }_{(i,j)}=\frac{180}{\pi }\arcsin \frac{{y}_{i}-{y}_{j}}{|({x}_{i}-{x}_{j},{y}_{i}-{y}_{j},{z}_{i}-{z}_{j})|},$$

where a node *i* is assumed to have the centroid coordinates (*x*_*i*_, *y*_*i*_, *z*_*i*_).

### Network flow model construction

Maximum Flow Minimum Cost (MFMC) is used to model fluid flow between two parallel walls (top inlet and bottom outlet) in a deforming granular material. Our intended application is the identification of preferential flow paths and their joint structure. As such, we formulate the *relative* capacity and cost through measures that capture the capacity of pores and throats to transmit fluid at a certain cost. If, instead, our application was to model actual fluid flow, then MFMC and the *actual* capacity and cost would have to satisfy the assumed physical flow behaviour (e.g. Stokes or Navier-Stokes) and be formulated accordingly. Such a representation is beyond the scope of this work.

In this work, MFMC is implemented using the LEMON library by Dezso *et al*.^59^ and formulated as follows. We denote ${\mathscr{G}}=(V,E,U,C,s,t)$ as the flow network. Vertices (nodes) *V* represent the pores and the set *E* contains the edges (links) that represent throats, as described in the previous section on pore network construction. Edges (*i*, *j*) ∈ *E* transmit a certain flow *f*_(*i*,*j*)_, limited by the capacities $U=\{{u}_{(i,j)}|(i,j)\in E\}$ and incurring costs $C=\{{c}_{(i,j)}|(i,j)\in E\}$. The capacity *u*_(*i*,*j*)_ is equal to the maximum number of units of flow *f*_(*i*,*j*)_ that (*i*, *j*) can transmit, with *c*_(*i*,*j*)_ representing the cost per flow unit. Flow is injected into ${\mathscr{G}}$ through an artificial source node *s*, connected (with infinite capacity and zero cost) to every inlet node. In the same way, a sink node *t* is connected to every outlet node. Optimization in the MFMC algorithm proceeds in two stages:

1. Find the maximum number of units of flow (*maximum flow*) |*f*|* that can be transmitted through ${\mathscr{G}}$, from source *s* to sink *t*, using Goldberg-Tarjan’s preflow push-relabel algorithm^60^, subject to the constraints:
  *f*_(*i*,*j*)_ ≤ *u*_(*i*,*j*)_ ∀ (*i*, *j*) ∈ *E* (capacity constraint)
  ∑_*i*∈*V*_ *f*_(*i*,*j*)_ = ∑_*k*∈*V*_ *f*_(*j*, *k*)_, *j* ≠ {*s*, *t*} (conservation of flow)
  ∑_*i*∈*V*_ *f*_(*i*, *t*)_ = ∑_*j*∈*V*_ *f*_(*s*, *j*)_ (conservation of flow)
2. Minimize the cost of transmitting |*f*|^*^ through ${\mathscr{G}}$ (*minimum cost*) using the primal network simplex algorithm^61,62^, subject to the constraints above.

The first stage of MFMC typically identifies the main bottleneck in the network, restricting |*f*|^*^ to the summed capacity of the edges in the bottleneck. The second stage then proceeds to optimize the transmission of |*f*|^*^ by finding the minimum cost pathways from the inlet to the bottleneck, and from the bottleneck to the outlet. The solution of the MFMC optimization problem provides (1) the maximum flow |*f*|^*^, capturing the total flow that can be ‘pushed’ through ${\mathscr{G}}$, (2) a percolating sub-network *P* of ${\mathscr{G}}$ that transmits |*f*|^*^ at minimum cost, and (3) the total cost of transmitting |*f*|^*^, defined as $|c{|}^{\ast }={\sum }_{(i,j)\in E}\,{f}_{(i,j)}{c}_{(i,j)}$. We plot the average cost of transmitting a unit of max flow (|*c*|^*^/|*f*|^*^) in Fig. 3(c) to reveal how the ease with which the material transmits flow evolves as the material deforms. Lastly, we note that the solution of the network flow model depends on the *relative* values of *u*_(*i*,*j*)_ and *c*_(*i*,*j*)_. That is, if (|*f*|^*^, |*c*|^*^, *P*) is the solution using capacities *u*_(*i*,*j*)_ and costs *c*_(*i*,*j*)_, then (*α*|*f*|^*^, *α*|*c*|^*^, *P*) is the solution for capacities *αu*_(*i*,*j*)_ and costs *αc*_(*i*,*j*)_ (*α* > 0).

In the remainder of this section, we present the definitions of capacity and cost. As a proxy for the relative transmission capacity of an edge in the network ${\mathscr{G}}$, representing a pore-throat-pore series, we assume equivalent cylindrical geometry for the pores and throats. Under the corresponding assumptions of tube flow, conductance is calculated using the Hagen-Poiseuille law:

3 $${C}_{\beta }=\frac{\pi {r}_{\beta }^{4}}{8\eta {L}_{\beta }},\,\beta \in \{i,j,{t}_{(i,j)}\},$$

where *i* and *j* denote the pores on either side of the connecting throat *t*_(*i*,*j*)_, *C* (m^3^ Pa^−1^ s^−1^) is the conductance, *r* (m) and *L* (m) are the respective radius and length the equivalent cylinder, and *η* (Pa s) is the dynamic viscosity of the fluid. For further details on the derivation of *L* and *r* for pores and throats we refer to the more extensive discussion in van der Linden *et al*.^54,55^. The conductance capacity function is given by the harmonic mean of the pore-throat-pore series:

4 $${u}_{(i,j)}=\frac{{L}_{i}+{L}_{{t}_{(i,j)}}+{L}_{j}}{\frac{{L}_{i}}{{C}_{i}}+\frac{{L}_{{t}_{(i,j)}}}{{C}_{{t}_{(i,j)}}}+\frac{{L}_{j}}{{C}_{j}}}.$$

For cost, we use a penalty value based on inertia. Capturing the intuition of ‘high cost’ for flow around a sharp bend, an edge (*i*, *j*) is given a high cost value if it aligns poorly with other edges connected to nodes *i* and *j*. To this end, we define the average angle of edge (*i*, *j*) with the *N*_*k*_ other edges (*i*, *k*) connecting to node *i* as:

5 $$\begin{array}{rcl}\overline{{\theta }_{i}} & = & \frac{180}{\pi {N}_{k}}\mathop{\sum }\limits_{k\mathrm{=1}}^{N}\,\arccos \frac{{{\bf{v}}}_{ij}\cdot {{\bf{v}}}_{ik}}{|{{\bf{v}}}_{ij}||{{\bf{v}}}_{ik}|}\\ {{\bf{v}}}_{ij} & = & [{x}_{j}-{x}_{i},{y}_{j}-{y}_{i},{z}_{j}-{z}_{i}],\\ {{\bf{v}}}_{ik} & = & [{x}_{k}-{x}_{i},{y}_{k}-{y}_{i},{z}_{k}-{z}_{i}],\end{array}$$

where (*x*_*i*_, *y*_*i*_, *z*_*i*_) are the coordinates of the centroid of the particle corresponding to node *i*. The average angle of edge (*i*, *j*) with every other edge connecting to node *j* is obtained by switching out *i* and *j* in (5). Using this expression, the inertia cost penalty is given by the harmonic mean:

6 $${c}_{(i,j)}={[\frac{2}{{\overline{{\theta }_{i}}}^{-1}+{\overline{{\theta }_{j}}}^{-1}}]}^{-1}$$

We take the inverse of the harmonic mean to ensure angles close to 180°, in alignment with (*i*, *j*), result in low cost.

### Classification

To perform the classification, we define a simple binary classifier, predicting inclusion or exclusion *χ*_(*i*,*j*)_ of a particular edge in the MFMC sub-graph *P*, i.e. *χ*_(*i*,*j*)_ = 1 if $(i,j)\in P$ and *χ*_(*i*,*j*)_ = 0 otherwise. The classifier is given by the logistic regression of *χ*_(*i*,*j*)_ on the four independent edge-level variables (features) ${X}_{1},\ldots ,{X}_{4}$ listed in Table 1, estimating the corresponding model parameters ${\beta }_{0},\ldots ,{\beta }_{4}$ by maximum likelihood in:

7 $$logit(p)={\beta }_{0}+{\beta }_{1}{X}_{1}+{\beta }_{2}{X}_{2}+{\beta }_{3}{X}_{3}+{\beta }_{4}{X}_{4},\,\,logit(p)={\rm{l}}{\rm{n}}(\frac{p}{1-p}),$$

where *p* is the probability that *χ*_(*i*,*j*)_ = 1. Other features were considered (capacity, throat length, vertical distance) but ultimately removed from the model due to multicollinearity given by other features in Table 1. Other (non-linear) classification methods were also considered, but did not provide noticeable improvement in classification performance. The features ${X}_{1},\ldots ,{X}_{4}$ are standardized to have zero-mean and unit-variance.

For previously unseen edges, a derived output of the logistic regression model in Eq. (7) is the probability that *χ*_(*i*,*j*)_ = 1. As such, a threshold is required to convert each probability prediction into the binary outcome: (*i*, *j*) ∈ *P* or (*i*, *j*) ∉ *P*. For probabilities above the threshold, we assume *χ*_(*i*,*j*)_ = 1 (positive case), while probabilities below the threshold are assumed to imply *χ*_(*i*,*j*)_ = 0 (negatives case). As is common, we calculate this threshold by simultaneously maximizing the fraction of true positives (*sensitivity*) and fraction of true negatives (*specificity*) using 20% of the 80% of edges reserved for model fitting in the sample with 21.54% axial strain used for Table 1. Sensitivity and specificity are competing objectives, as increasing the threshold increases the likelihood of correctly predicting all negative cases, while decreasing the likelihood of predicting all positive cases. For this particular set of edges, we obtain a threshold of 0.73.

### Permeability

The approximated numerical permeability is calculated using Stokes’ law and the OpenPNM Python package^63^. Using the conductances *C*_(*i*,*j*)_ calculated in Eq. (4), the conservation of mass prescribes:

8 $$\sum _{i\to j}\,{Q}_{(i,j)}=\sum _{i\to j}\,{C}_{(i,j)}({P}_{i}-{P}_{j})=0,$$

where *Q*_(*i*,*j*)_ is the flowrate (m^3^ s^−1^) between nodes *i* and *j* and *P*_*i*_ is the pressure (Pa). We express Eq. (8) as linear system of equations, prescribing *P*_*i*_ = 10 Pa for all inlet nodes *i*, and *P*_*j*_ = 9 Pa for all outlet nodes *j*. We obtain the pressures at all other nodes by solving the corresponding matrix-vector equation. Lastly, the net outlet flow *Q*_our_ is inserted into Darcy’s law to calculate permeability:

9 $$k=\frac{\eta L{Q}_{out}}{A\Delta P},$$

where *L* is the height of the sample, *A* is the cross-sectional area, *η* = 0.001002 Pa s is the fluid dynamic viscosity, and Δ*P* = 1 is the pressure difference over inlet and outlet nodes. For spherical particle assemblies, Stokes flow has been shown to agree moderately well with physical measurements^64^.

### Shortest paths and network centrality

We define *d*_(*i*,*j*)_ as the length of the shortest path distance from node *i* to node *j*, minimizing the number of traversed edges. Shortest paths are calculated using Dijkstra’s algorithm^65^. To assess network connectivity, we compute the average closeness, betweenness and information centrality. Closeness centrality for a node *i* is given by^66^:

10 $${C}_{i}^{C}=(|V|-1){[\sum _{j\ne i}d(i,j)]}^{-1},$$

where |*V*| is the number of nodes. Closeness centrality is a multi-scale measure of connectivity, capturing the degree of connectivity of nodes to all other nodes. Betweenness centrality further characterizes this connectivity by computing the fraction of shortest paths passing through a particular edge^67^:

11 $${C}_{(i,j)}^{B}=\frac{1}{2}(|V|-\mathrm{1)(|}V|-\mathrm{2)}\,\sum _{k\in {V}_{{\rm{in}}},\,l\in {V}_{{\rm{out}}}}\,\frac{{\sigma }_{kl}(i,j)}{{\sigma }_{kl}},$$

where *V*_in_ and *V*_out_ are the respective inlet and outlet nodes, *σ*_*kl*_ is equal to the total number of shortest paths between node *k* and *l*, and *σ*_*kl*_(*i*, *j*) is the number of shortest paths between *k* and *l* that pass through edge (*i*, *j*). We opt for the specific case where *k* ∈ *V*_in_ and *l* ∈ *V*_out_ (as opposed to *k*, *l* ∈ *V*) to assess the degree of path localization in the direction of flow.

Shortest path distance, closeness centrality and betweenness centrality quantify connectivity by assuming quantities traverse the network solely along non-diverging shortest paths. In a connected and saturated porous medium, however, fluid will flow along non-preferential paths too. To this end, information centrality quantifies connectivity through electrical current flow paths, which, similar to electrical current, may diverge, converge and follow non-preferential paths^68^. We calculate information centrality for every node *i* as^69^:

12 $${C}_{i}^{I}=(|V|-1){[\sum _{j\ne i}{p}_{ij}(i)-{p}_{ij}(j)]}^{-1},$$

where *p*_*ij*_(*k*) is the absolute potential of node *k* if a single unit of current is injected at node *i* and leaves the network at node *j*. Given this unit supply, the absolute potential is calculated by solving the linear set of equations given by Ohm’s and Kirchoff’s potential laws:

13 $$\sum _{i\to j}\,{x}_{(i,j)}=\sum _{i\to j}\,{C}_{(i,j)}({p}_{i}-{p}_{j})=0,$$

where *x*_(*i*,*j*)_ is the current and *C*_(*i*,*j*)_ is the conductance. We use (4) for the conductance, in which case (13) is equivalent to Stokes law’ (8) with a unit flow supply at node *i* and sink at node *j*. The term *p*_*ij*_(*i*) − *p*_*ij*_(*j*) in (12) is the effective resistance (or pressure difference) acting as an alternative to the shortest path distance used in (10).

## Supplementary information

Supplementary material

## Data Availability

The datasets generated during and/or analyzed during the current study are available from the corresponding author on reasonable request.
