# Supplementary material for: Preferential flow pathways in a deforming granular material: self-organization into functional groups for optimized global transport
Source: Sci Rep. 2019 Dec 3;9:18231. doi: 10.1038/s41598-019-54699-6 (PMC6890641; doi:10.1038/s41598-019-54699-6)
Supplement: Supplementary file 1 — Supplementary material [file 41598_2019_54699_MOESM1_ESM.pdf]

Preferential flow pathways in a deforming granular material: self-organization into functional groups for optimized global transport

Joost H. van der Linden[1], Antoinette Tordesillas[2,*], and Guillermo A. Narsilio[1]

[1]Department of Infrastructure Engineering, The University of Melbourne, Australia
[2]School of Mathematics and Statistics & School of Earth Sciences, The University of Melbourne, Australia
[*]Corresponding author; atordesi@unimelb.edu.au

1 Supplementary information

1.1 Proof of principle: Demonstrate utility of the network flow model against observed data

To assess the degree to which our network flow model captures physical fluid flow behavior, the output is compared to Stokes flow and Navier-Stokes flow for a range of relatively homogeneous sphere packings with varying void ratio. Results are shown in Figure 1. The fifteen packings by Song et al.[1] were generated by isotropically compressing monodisperse particles to a mechanically jammed state at varying porosities, using a Hertz-Mindlin contact model and periodic boundary conditions. The six assemblies from van der Linden et al[2] also originated from a discrete-element simulation, consisting of a gravity deposition followed by compression. The data is further separated into three monodisperse packings ($\alpha = 0\%$) and three packings with mild polydisersity ($\alpha = 25\%$, corresponding to the uniform particle size distribution $U(0.5-0.125, 0.5+0.125)$ mm). Lastly, we compare our results for a series of real sphere packings by Aste et al.[3–5]. Aste and co-authors used x-ray computed tomography to measure particle centroids and radii of assemblies of glass and acrylic beads. Samples were prepared by pouring the beads into a cylindrical container at different speeds and by using tapping (seven samples from[4]), or by submerging the samples and subjecting the assemblies to flow pulses from below (three samples from[5]) to vary density.

As expected for monodisperse and mildly polydisperse sphere packings, we observe that the radius-normalized permeability curves collapse in Figure 1. Furthermore, permeability increases with void ratio, for both Stokes- and Navier-Stokes flow. We attribute the slight consistent overestimation of permeability by the Stokes equation to the idealized (cylindrical) pore and throat geometry used to calculate conductance. Our Navier-Stokes simulations exhibit low Reynolds' numbers ($Re << 1$), hence inertia effects are not expected to decrease permeability for Navier-Stokes. Narsilio et al.[6] showed that the Navier-Stokes permeability provides a reasonable numerical approximation of the experimentally measured permeability for sphere packings. We conclude that, under these assumptions, Stokes flow provides a reasonable approximation of macro-scale flow behavior.

Maximum flow shows a similar increase with void ratio, though slightly reduced, compared to permeability. Indeed, $\eta|f|^*/A/r$ and k_s/r^2 are highly correlated (Pearson $r = 0.99$, $p < 10^{-12}$, using square root transformation to ensure normality). For more porous sphere assemblies, pore size and throat area are larger, increasing, in turn, the conductance capacity values. In the absence of any inhomogeneities (i.e. bottlenecks), higher capacities result in higher maximum flow, as shown. In contrast, the normalized total cost is strongly inversely correlated with permeability ($r = -0.99$, $p < 10^{-12}$). This behavior captures the intuition that it is 'cheaper' to pass through a unit of maximum flow between inlets and outlets for more porous assemblies. Such a decrease in cost is attributed to larger pores and throats, resulting in increased availability of more direct flow routes that require both fewer and better aligned edges in the pore network. Inserts on the bottom right of Figure 1 illustrate two such examples in 2D. Flow pathways for the low void ratio example are tortuous and, due to smaller pores, require more edges to cross from top to bottom. Edges in the pathways through the loose, high void ratio sample, on the other hand, are generally longer and better aligned, resulting in smaller costs.

1.1.1 Comparison at the micro-scale

Having compared our network flow model results with Stokes flow permeability at the macro-scale, the question remains how well maximum flow at minimum cost compares with flow rates at the micro-scale. Figure 2 shows the edge flow for maximum flow (2a) and maximum flow at minimum cost (2b), plotted against the Stokes flow rate, for one of the assemblies (with void ratio 0.7) by Song et al[1]. Marginal distributions are plotted along the axis, and contour lines indicate the density of values in the joint distribution. The square root transformation is used to ensure normality and reveal the bimodal nature of the maximum flow distribution. Note that the units of flow in MFMC are irrelevant, and we are mainly interested in the correlation with physical flow.

Unsurprisingly, correlation between maximum flow and Stokes flow at the edge-level is not as strong as the macro-scale correlations in Figure 1. Maximum flow only accounts for capacity, pushing as much flow through the network as possible, ignoring altogether the effect of cost and preferential routes through the network. Indeed, when the inertia cost function is used to calculate maximum flow at minimum cost, the correlation coefficient improves from 0.58 to 0.75. The two peaks in the bimodal normal distribution of $\sqrt{f_{i,j}}$ in P^0 and $\sqrt{f_{i,j}}$ in P correspond to the two most common throat configurations, as shown by the two inserts in the middle of Figure 2. These configurations have a defined throat area, resulting in two frequently occurring conductance values, that, in turn, show up as two peaks in the edge flow distribution. The improvement of the linear correlation coefficient when accounting for cost is consistent across all datasets, as shown in Table 1. All corresponding p-values were found to be smaller than $2 \cdot 10^{-16}$.

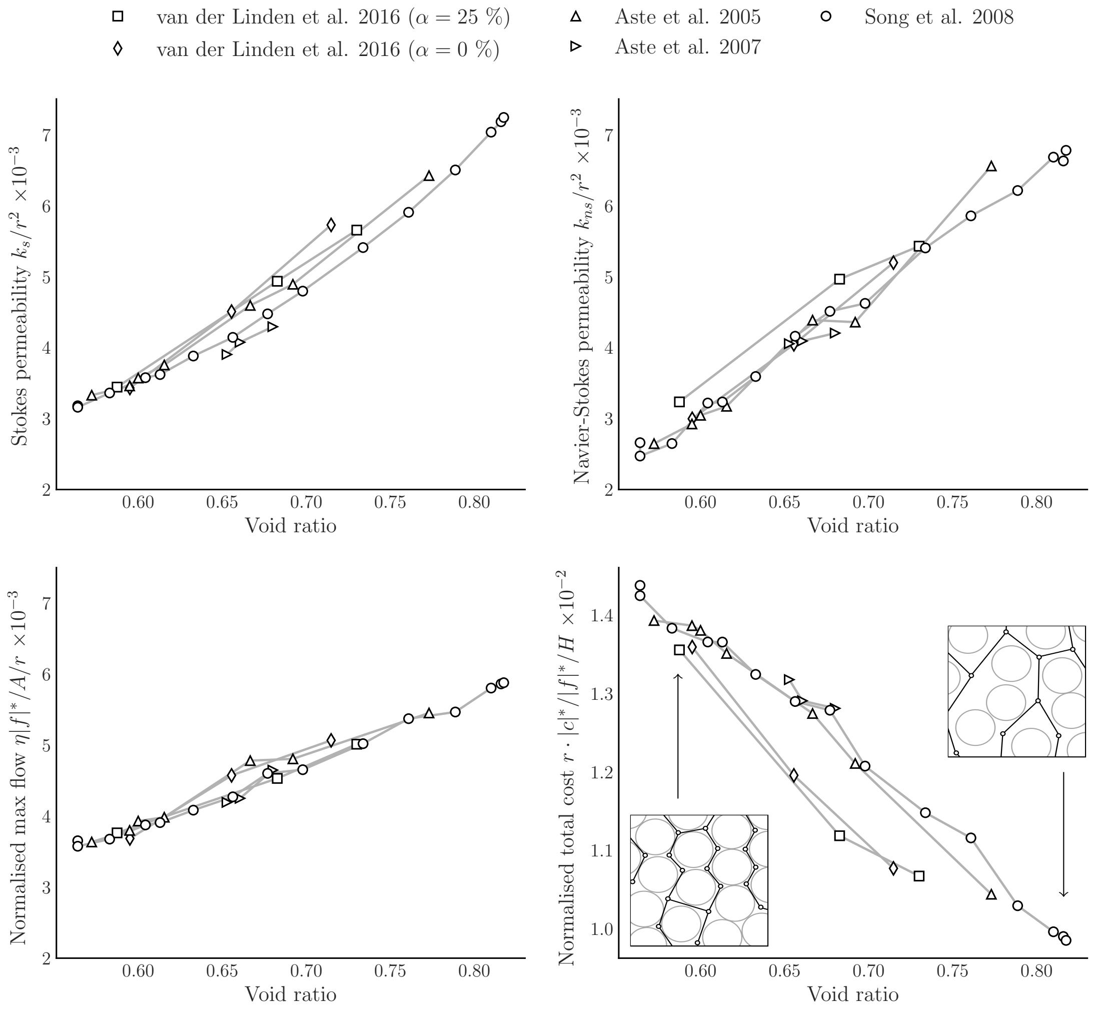

Figure 1. *Maximum flow and total cost show good agreement with the permeability trend in samples with varying void ratio.* Comparison of the permeability from Stokes equation (network simulation) and Navier-Stokes equation (finite element simulation), with the maximum flow value (normalized by dynamic viscosity η, cross-sectional area A and particle radius r) and total cost (normalized by particle radius, maximum flow $|f|^*$ and sample height H). Inserts on the bottom right illustrate two (2D equivalent) pore networks at different void ratios.

Table 1. Pearson correlation coefficients for the relationship of the (square-root transformed) Stokes flow rate, with Max flow and Max flow at min cost, averaged across all samples in the dataset.

	Average pearson correlation coefficient	
Samples	Max flow	Max flow at min cost
Song et al.[1]	0.49	0.74
van der Linden et al.[2]	0.54	0.77
Aste et al.[3,4]	0.51	0.74
Aste et al.[5]	0.49	0.73

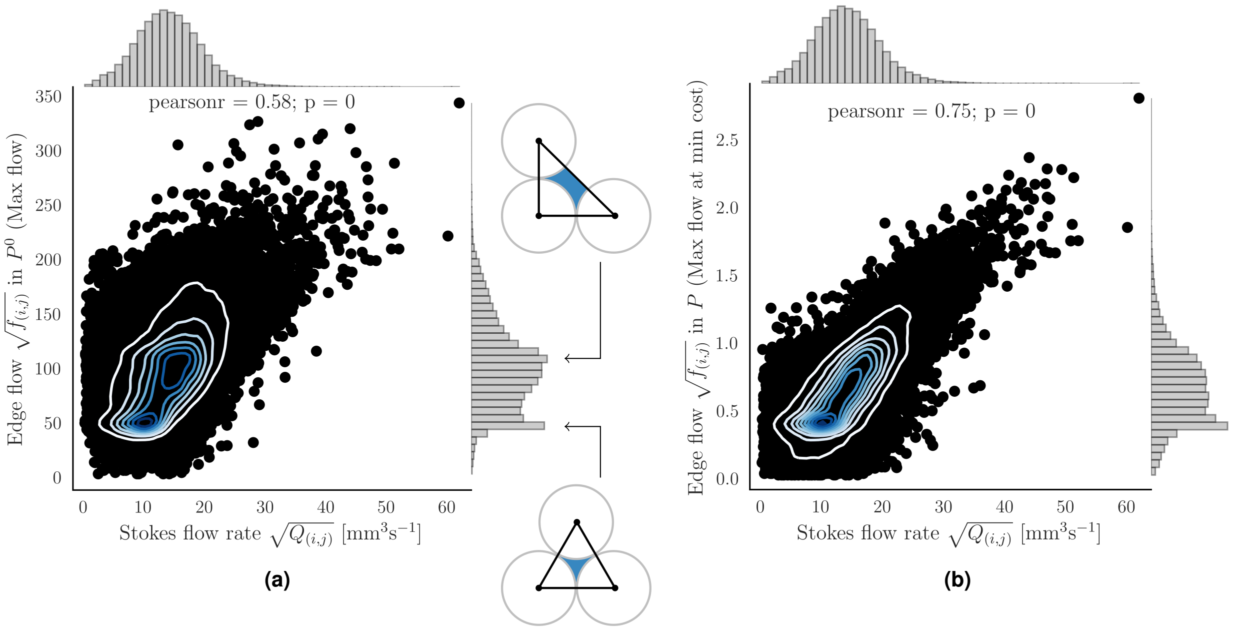

Figure 2. Comparison of the square-root transformed Stokes flow rate [mm^{3}s^{-1}] with the edge flow values of max flow in the max flow network P^0 and max flow at min cost in the corresponding network P, for the sample with void ratio 0.7 by Song et al.[1].

References

1. Song, C., Wang, P. & Makse, H. a. A phase diagram for jammed matter. *Nat.* **453**, 629–632 (2008).
2. van der Linden, J. H., Narsilio, G. A. & Tordesillas, A. Machine learning framework for analysis of transport through complex networks in porous, granular media: A focus on permeability. *Phys. Rev. E* **94**, 022904 (2016).
3. Aste, T., Saadatfar, M., Sakellariou, A. & Senden, T. J. Investigating the geometrical structure of disordered sphere packings. *Phys. A: Stat. Mech. its Appl.* **339**, 16–23 (2004).
4. Aste, T., Saadatfar, M. & Senden, T. Geometrical structure of disordered sphere packings. *Phys. Rev. E* **71**, 061302 (2005).
5. Aste, T. *et al.* An invariant distribution in static granular media. *Europhys. Lett.* **79**, 24003 (2007).
6. Narsilio, G., Buzzi, O., Fityus, S., Yun, T. & Smith, D. Upscaling of navier–stokes equations in porous media: Theoretical, numerical and experimental approach. *Comput. Geotech.* **36**, 1200–1206 (2009).
